# TNBC invasion: downstream of STAT3

**DOI:** 10.18632/oncotarget.15259

**Published:** 2017-02-10

**Authors:** James G. Jackson, Guillermina Lozano

**Affiliations:** Department of Genetics, The University of Texas MD Anderson Cancer Center, Houston, TX, USA

**Keywords:** breast cancer, ChIP, STAT3, mutant p53

Breast cancer arises from luminal or basal cells lining the mammary duct and has been classified into four subgroups based on gene expression: luminal A, luminal B, HER2 enriched, and basal-like [1]. The majority of the basal-like tumors lacks expression of hormone receptors ER, PR and HER2 and has thus been termed triple negative breast cancer (TNBC). TNBC has a particularly poor prognosis due to insensitivity to therapies that target the receptors they lack. Genomic sequencing of TNBC has revealed a catalogue of changes, the most common of which occur in *TP53* (80%), *PTEN* (35%), *RB1*, and *BRCA1* genes [2]. In addition to genomic studies, our understanding of TNBC was expanded in other studies, including a large scale shRNA screen on basal-like cell lines that identified JAK2/STAT3 as an essential pathway [3]. Inhibition of JAK2 decreased cell number and blocked growth of xenografts [3].

The McDaniel et al study [4] furthered this analysis by performing STAT3 Chromatin Immunoprecipitation (ChIP) followed by massively parallel sequencing (ChIP-seq) on five basal-like TNBC cell lines (two of which [MDA-MB-231 and MDA-MB-468] were used in the above shRNA screen that identified the JAK2/STAT3 pathway) to identify the transcriptional program driven by STAT3 activation. The authors also performed STAT3 ChIP-seq on two primary TNBCs, and, importantly, found a high degree of correlation to the results from cell lines [4].

STAT3-specific regulation of gene expression was determined by gene expression changes in TNBC cell lines after STAT3 knockdown. STAT3 binding sites were identified within 50KB of 64% and 39% of genes differentially expressed after STAT3 knockdown in HCC70 and MDA-MB-231, respectively. Twenty-two genes were regulated by STAT3 in both cell lines, but this small signature was not enriched for any biological processes suggesting inherent heterogeneity within the cell lines. However, both cell lines were enriched for genes involved in invasion and migration, and integration of all datasets across five cell lines (the two above plus HCC1143, MDA-MB-157, MDA-MB-468) revealed significant enrichment in genes involved in invasive processes. STAT3 specific knock down in two of the cell lines impaired invasion, but did not affect viability or proliferation [4].

These data provide a comprehensive characterization of gene regulation by STAT3, and show a role for STAT3 in promoting invasive properties in TNBC. Data from TNBC cell lines and tumors show regulation of genes unique to individual cell lines and common among them all. What's not in common could be important for driving heterogeneity among TNBC tumors, and also begs the question about what other transcription factors are cooperating to drive a STAT3 program. Conversely, it is possible that only the overlapping genes drive TNBC although this overlapping dataset does not have an invasive signature.

One area of interest for the future concerns the mechanism of STAT3 activation in the cell lines, which was not determined in this study. While the correlation of binding sites observed in actual tumors was a powerful validation for the findings in cell lines, STAT transcription factors can be regulated by a plethora of upstream ligands, receptors and kinases, and the primary route of STAT activation in the TNBC tumors and cell lines in this study remains unknown. In the cell lines examined, STAT3 activation was, at least in part, due to components of serum in the media. The IL-6/JAK2/STAT3 pathway is required for growth of basal-like breast cancer cells (including MDA-MB-231 and MDA-MB-468) implicating IL-6 and JAK2 as possible regulators [3].

In addition to JAK2/STAT3, p53 also plays a critical role in TNBC. Not surprisingly, p53 alterations occur in all five TNBC cell lines studied, four of which have p53 missense mutations (one has a frame shift mutation). A large body of data indicates that mutant p53 proteins have additional activities that fuel metastatic properties such as invasion and motility [5]. Thus, TNBC tumor cells appear to have at least two pathways that lead to their aggressive behavior, activation of JAK2/STAT3 and mutant p53. As noted above, the JAK2/STAT3 pathway was found to be essential for viability in TNBC cell lines [3]. Mutant p53 depletion in breast cancer cells (MDA-MB-231 cells with p53R280K and MDA-MB-468 with p53R273H) in 3D culture leads to phenotypic reversion to more normal, differentiated structures with hollow lumens suggesting that cells with mutant p53 may be “addicted” to mutant p53 [6]. Data from various tumor types, such as lymphoma, suggest that tumor cells are “addicted” to mutant p53 for survival [7]. Thus, combination therapies to target both of these pathways may be more beneficial to the patient in lethal, metastatic TNBC.

STAT3 binding activity in breast cancer cell lines derived from other subtypes and cultured in serum was not examined. How much of the profile described here is unique to the TNBC cell lines and tumors? One possible extension of these studies that were focused on constitutive STAT3 activation in predominantly *TP53* mutant TNBC, is the potential role for STAT3 in other subtypes of breast cancer that are predominately *TP53* wild type. While STAT3 may not be constitutively active in these tumors, it can be strongly activated by cytokines induced in tumor cells (including breast) made senescent by genotoxic chemotherapy [8, 9]. In various tumor types, this mode of STAT3 activation can promote tumor growth, survival, stem cell enrichment, invasive/metastatic properties, and, ultimately, relapse [9]. In light of data presented in McDaniel et al study, one might hypothesize that secretion of STAT3 activating ligands by treated, responding tumors that lacked constitutive activation of STAT3, could promote or contribute to relapse and distal recurrence.

In sum, McDaniel et al present an unbiased, genomic scale characterization of STAT3 transcriptional regulation in TNBC. These data will be an invaluable resource for future studies examining STAT3 regulation across different tumor types and model systems.

## References

[1] Perou CM (2000). Nature.

[2] TCGA-Network (2012). Nature.

[3] Marotta LL (2011). J Clin Invest.

[4] McDaniel JM (2017). Oncotarget.

[5] Brosh R (2009). Nat Rev Cancer.

[6] Freed-Pastor WA (2012). Cell.

[7] Alexandrova EM (2015). Nature.

[8] Jackson JG (2012). Cancer cell.

[9] Rao SG (2016). Trends in Cancer.

